# Functional tooth restoration utilising split germs through re-regionalisation of the tooth-forming field

**DOI:** 10.1038/srep18393

**Published:** 2015-12-17

**Authors:** Naomi Yamamoto, Masamitsu Oshima, Chie Tanaka, Miho Ogawa, Kei Nakajima, Kentaro Ishida, Keiji Moriyama, Takashi Tsuji

**Affiliations:** 1 Department of Maxillofacial Orthognathics, Tokyo Medical and Dental University, Bunkyo-ku, Tokyo, 113-8510, JAPAN; 2 Department of Biological Science and Technology, Graduate School of Industrial Science and Technology, Tokyo University of Science, Noda, Chiba, 278-8510, JAPAN; 3 Department of Oral Rehabilitation and Regenerative Medicine, Graduate School of Medicine, Dentistry and Pharmaceutical Sciences, Okayama University, Okayama, 700-8525, JAPAN; 4 Research Institute for Science and Technology, Tokyo University of Science, Noda, Chiba, 278-8510, JAPAN; 5 Organ Technologies Inc., Tokyo, 108-0074, JAPAN; 6 RIKEN Center for Developmental Biology, Kobe, Hyogo, 650-0047, JAPAN; 7 Department of Clinical Pathophysiology, Tokyo Dental College, Chiyoda-ku, Tokyo, 101-0061, JAPAN; 8 Department of Physics and Mathematics, College of Science and Engineering, Aoyama Gakuin University, Sagamihara, Kanagawa, 252-5258, JAPAN

## Abstract

The tooth is an ectodermal organ that arises from a tooth germ under the regulation of reciprocal epithelial-mesenchymal interactions. Tooth morphogenesis occurs in the tooth-forming field as a result of reaction-diffusion waves of specific gene expression patterns. Here, we developed a novel mechanical ligation method for splitting tooth germs to artificially regulate the molecules that control tooth morphology. The split tooth germs successfully developed into multiple correct teeth through the re-regionalisation of the tooth-forming field, which is regulated by reaction-diffusion waves in response to mechanical force. Furthermore, split teeth erupted into the oral cavity and restored physiological tooth function, including mastication, periodontal ligament function and responsiveness to noxious stimuli. Thus, this study presents a novel tooth regenerative technology based on split tooth germs and the re-regionalisation of the tooth-forming field by artificial mechanical force.

Organs originate from their respective organ germs through reciprocal epithelial-mesenchymal interactions between the immature epithelium and the mesenchyme in the developing embryo^1^,^2^,^3^,^4^. Organ development, which relies on inductive properties such as regional and genetic specificity, is regulated principally by a developmental mechanism based on epithelial-mesenchymal interactions that involve signalling molecules and transcription factor pathways^5^. It is well known that signalling molecules, including fibroblast growth factors (FGFs), hedgehog, Wnts and bone morphogenetic proteins (BMPs), play essential roles in epithelial-mesenchymal interactions during organogenesis^1^,^2^. These molecular factors are also involved in the characteristic morphogenesis patterning during organ development and contribute to determining the size of the three-dimensional organ, through macro-patterning, and the complex morphology of the organ, through micro-patterning^2^. Structural patterning, such as digits in limbs, feathers in skin and cusps in teeth, is based on a reaction-diffusion model that determines the expression of distinct molecules, including FGF4 and BMPs, during organogenesis^6^,^7^,^8^. Thus, tissue formation and morphogenesis are spatiotemporally regulated during developmental processes by gradients of morphogens^9^ and by a reaction-diffusion model that involves the expression of multiple related genes^10^.

During early craniofacial development in mice, tooth-forming fields are specified during embryonic days (ED) 10–11 through the expression of homeobox genes (such as Msx1 and Msx2) and secreted molecules (including FGFs and BMPs)^3^,^7^,^11^. The tooth-forming field is also regulated by a reaction-diffusion mechanism consisting of the spatiotemporal patterning of the expression of *Lef1*, an activator, and of *Ectodin*, an inhibitor^12^. Subsequently, the oral epithelium becomes invaginated in the mesenchymal region, and a tooth germ is formed by the aggregation of neural crest-derived mesenchymal cells. A transient epithelial signalling centre, designated as the primary enamel knot, expresses several signalling molecules, including SHH, Wnt, BMPs and FGFs, and is thought to coordinate individual tooth cell fates and epithelial-mesenchymal interactions during ED 13.5–14.5^1^. Tooth morphology, including tooth size and tooth cusp number, is determined in the tooth-forming field by the expression of specific genes in the immature oral epithelium and the neural crest-derived mesenchyme of the embryonic jaw^3^. The macro-patterning of tooth size can be modelled by the reaction-diffusion mechanism that is regulated by the signalling molecules in the dental mesenchyme^13^. Furthermore, micro-patterning of the number and position of the tooth cusps is thought to involve the expression of FGF4 at secondary signalling centres, which are designated as secondary enamel knots^7^,^14^. Thus, the specific localisation (or regionalisation) of endogenous inhibitors and mesenchymal activators, and therefore the balance between these, generates reaction-diffusion waves and is essential for tooth morphogenesis^14^,^15^,^16^.

Congenital tooth loss caused by cleft lip and/or cleft palate, ectodermal dysplasia, Down syndrome or non-syndromic diseases is one of the most common developmental anomalies in humans^17^. These patients exhibit fundamental impairments including occlusal dysfunction, aesthetic disorders and social problems that begin at a young age^18^. Acquired tooth loss in adults from dental caries, periodontal disease or trauma can be managed using conventional dental treatments involving artificial teeth, such as fixed dental bridges, removable dentures and dental implants^19^,^20^. Although these artificial dental therapies have been widely applied to correct tooth loss, further technological improvements based on biological findings are expected to also restore physiological tooth function^21^. Previous studies of autologous tooth germ transplantation, showing that this process prevented immunological rejection after transplantation, have reported successful tooth eruption into the oral cavity and restoration of physiological tooth function^22^,^23^,^24^. Furthermore, another method of functional tooth replacement has been demonstrated and is attractive: bioengineered tooth germs have been reconstructed using transplanted tooth-germ-derived stem cells^25^,^26^. However, the available tooth germs in humans are limited by the number and the developmental stage of individual tooth germs. Based on recent biological findings, we anticipate the development of a novel bioengineering technology that can increase the number of tooth germs.

In this study, we developed a novel mechanical ligation method for splitting tooth germs through spatiotemporal regulation of the reaction-diffusion waves in the tooth-forming field. Each of the split tooth germs developed multiple underlying signalling centres and eventually developed into natural teeth. After transplantation, the split teeth successfully erupted into a region of tooth loss and restored physiological tooth functions, including mastication, periodontal ligament function and appropriate responses to noxious stimuli. Thus, this study reports a technological development based on biological mechanisms that can increase the number of available tooth germs for tooth germ transplantation.

## Results

### Generation of split tooth germs by a mechanical ligation method.

**Figure 1:**
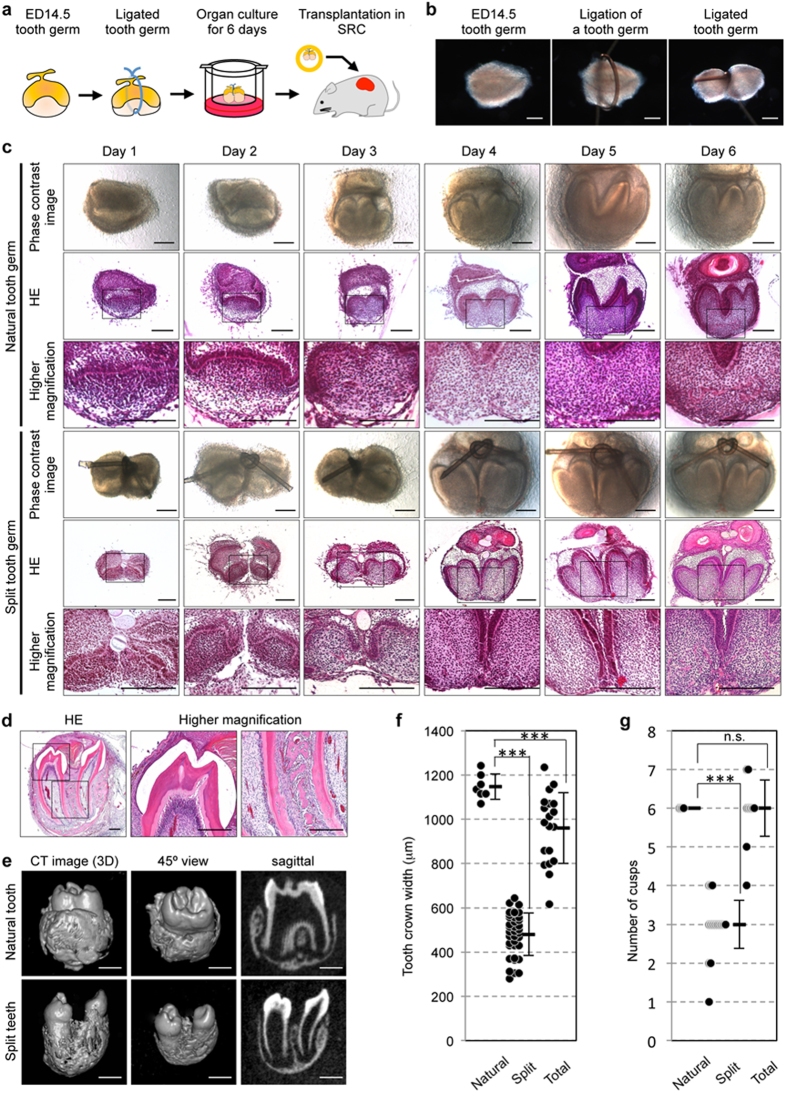
Generation of a split tooth germ. (**a**) Schematic representation of ligation of a tooth germ and transplantation into a SRC. (Illustration by N.Y.). (**b**) Photograph of an ED14.5 tooth germ (left), the creation of a noose from a single strand of 8–0 nylon thread (middle), and the tightened noose around the tooth germ (right). Scale bar, 100 μm. (**c**) Time course images of a natural tooth germ and a ligated tooth germ in *in vitro* organ culture. Phase-contrast images (upper) and HE section images (lower) of each tooth germ and each split tooth germ. Scale bar, 100 μm. (**d**) Histological analysis of the split teeth on day 30 after SRC transplantation. Higher magnification images of the crown area (centre) and the periodontal tissue area (right). Scale bar, 200 μm. E, enamel; D, Dentin; AB, Alveolar bone; PDL, periodontal ligament. (**e**) Micro-CT images of the external surface area (left), 45° view (centre) and cross section (right) of a natural tooth (upper images) and split teeth (lower images) at 30 days after SRC transplantation. Scale bar, 500 μm. (**f**) The crown width of natural teeth (Natural), each split tooth (Split) and the total width of split teeth (Total) at 30 days after SRC transplantation. The data are presented as the mean ± S.D.; n = 7 for natural teeth, n = 19 for split teeth. ^***^p < 0.001 (Dunnett’s test). (**g**) The number of cusps in natural teeth, the number of cusps in each split tooth and the total number of cusps in the split teeth at 30 days after SRC transplantation. The data are presented as the mean ± S.D.; n = 7 for natural teeth, n = 16 for split teeth. ^***^p < 0.001, n.s., not significant (Steel’s test).

To generate multiple organs from a developing organ germ by applying external force, we developed a mechanical ligation method for splitting tooth germs. We dissected molar tooth germs from the mandibles of ED14.5 mice as previously described^26^. These tooth germs were in the early tooth developmental stage (the cap stage), in which a signalling centre designated the primary enamel knot develops in the epithelial tissue^1^. A single tooth germ was longitudinally ligated through the central portion using thin nylon thread. However, the tooth germ was not completely split into two during this mechanical ligation method (Fig. 1a,b). Eighty-two per cent of ligated tooth germs (729/889) separated into two tooth germs after 6 days in an *in vitro* organ culture. The two split tooth germs were equivalent to two tooth germs that are associated during natural tooth development, except that the crown width of each split tooth germ was about half that of natural tooth germs (Fig. 1c). Additionally, ligation of tooth germs during the early developmental stage at ED13.5 or during the late developmental stage at EDs 15.5–18.5 was observed for incompletely split tooth germs, which exhibited fundamental problems: a low frequency of tooth formation and irregularity of tooth tissue structures, respectively. Only tooth germs that were ligated at ED14.5 were able to develop into correctly divided tooth germs that resulted in natural tooth development (Supplementary Fig. 1a,b).

We analysed tooth formation from the tooth germs that were split at ED14.5 in a subrenal capsule *in vivo* (Fig. 1a). Histological and micro-CT analyses indicate that 73.7% of the split tooth germs (28/38) successfully developed hard tissues (including enamel, dentin and alveolar bone) within 30 days of transplantation, generated two teeth with the correct structures of complete molars, and properly formed partitioned periodontal tissue and surrounding alveolar bone (Fig. 1d,e and Supplementary Fig. 1c,d). The mean crown widths of natural teeth, the widths of each split tooth at 30 days after transplantation, and the total widths of the split teeth were 1147.8 ± 58.0 μm, 480.0 ± 95.3 μm and 960.1 ± 159.6 μm, respectively (Fig. 1f). Furthermore, the number of cusps in natural teeth, the number of cusps in each split tooth at 30 days after transplantation, and the total number of cusps in the split teeth were 6.0 ± 0.0, 3.0 ± 0.6 and 6.0 ± 0.7, respectively (Fig. 1g). These results indicate that the ED14.5 tooth germ, which created a primary enamel knot at the tooth developmental stage, can be split by an external force applied via a mechanical ligation method and can subsequently develop correctly into multiple teeth.

### Developmental process and gene expression patterns of the split tooth germ.

**Figure 2:**
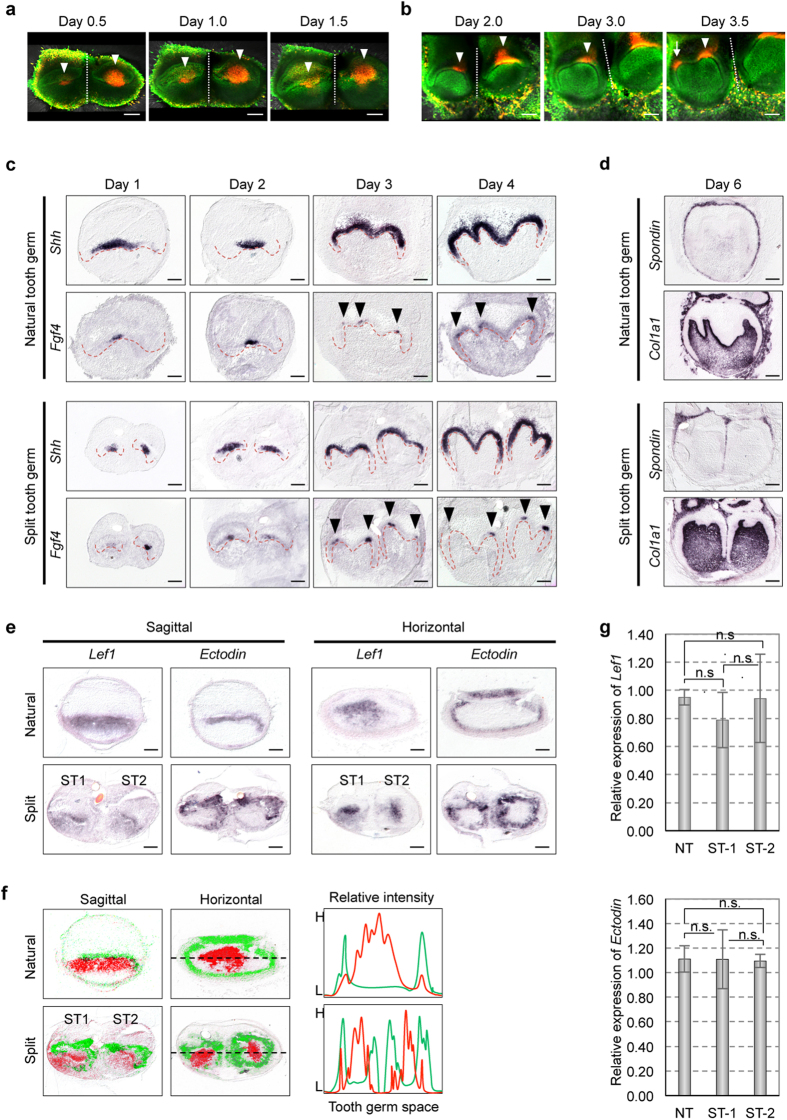
Morphogenesis of a split tooth germ. (**a**,**b**) Snapshot images of a time-lapse observation of a split tooth germ from a transgenic mouse embryo using a confocal laser microscope: the early developmental stage showing expression of the primary enamel knot (**a**), and the late developmental stage showing expression of the secondary enamel knot (**b**). Red denotes the growth-arrested regions. Dotted lines indicate the ligation position. Arrowheads and arrows indicate the primary enamel knots and the secondary enamel knot, respectively. Scale bar, 100 μm. (**c**) *In situ* hybridisation analysis of *Shh* and *Fgf4* expression in ED14.5 natural tooth germs (upper) and split tooth germs (lower) after 1, 2, 3 and 4 days of organ culture. Dotted lines indicate the boundaries between the epithelium and the mesenchyme. The arrowheads indicate the secondary enamel knots. Scale bar, 100 μm. (**d**) *In situ* hybridisation analysis of the *Spondin* and *Col1a1* expression profiles in ED14.5 natural tooth germs (upper) and split tooth germs (lower) after 6 days of organ culture. Scale bar, 100 μm. (**e**) *In situ* hybridisation analysis of the *Lef1* and *Ectodin* expression profiles of sagittal (left) and horizontal (right) sections of ED14.5 natural tooth germs (upper) and split tooth germs (lower) after 1 day of organ culture. Scale bar, 100 μm. ST, split tooth germ. (**f**) False collar overlays of *Lef1* (red) and *Ectodin* (green) expression patterns in ED14.5 natural tooth germs (upper) and split tooth germs (lower) in sagittal (left) and horizontal (centre) sections. Anterior-posterior (AP) profiles (dashed line) highlight the progressive appearance of *Lef1* (red) and *Ectodin* (green) in the graphs shown on the right. (**g**) The expression levels of *Lef1* (upper) and *Ectodin* (lower) in the natural tooth germs (NT) and each split tooth germ (ST-1 and ST-2) were quantified by real-time PCR. The data is presented as the mean ± S.D.; n = 3 for natural tooth germs, n = 5 for split tooth germs. n.s., not significant (Tukey test).

**Figure 3:**
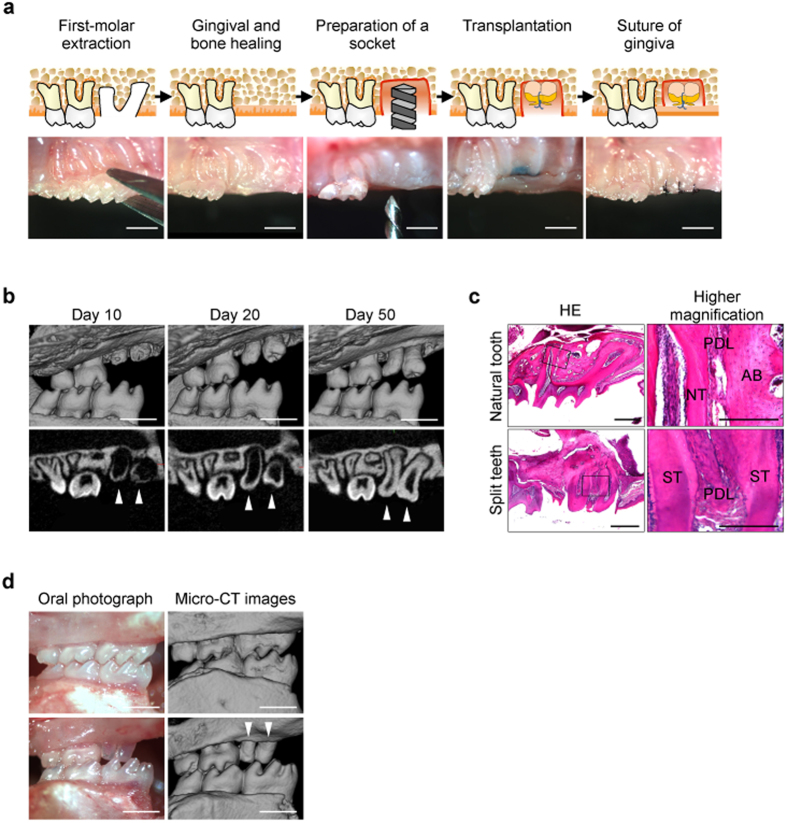
Transplantation of a split tooth germ. (**a**) Schematic representation (top; illustration by N.Y.) and photographs (bottom) of split tooth germ transplantation into adult jaw bones. Scale bar, 500 μm. (**b**) Micro-CT images of split teeth during the eruption process in three-dimensional (upper) and sagittal (lower) sections. Scale bar, 500 μm. (**c**) Histological analysis of a normal tooth (upper) and split tooth (lower), shown at higher magnification on the right. Scale bar, 500 μm. ST, split tooth; NT, natural tooth; AB, alveolar bone; PDL, periodontal ligament. (**d**) Images (left) and micro-CT images (right) of the occlusion of normal (upper) and split (lower) teeth. Scale bar, 500 μm.

To investigate the developmental process of the split tooth germ, we performed time-lapse live imaging analysis using transgenic mouse embryos to visualise the cell cycle *in vivo* by monitoring fluorescence in cells in the S/G2/M phases (green nuclei) and those in the G0/G1 phase (red nuclei)^27^,^28^. During tooth development, the formation and function of the enamel knots are related to the spatial regulation of growth arrest in the dental epithelium^29^,^30^. The prospective primary enamel knots, which were labelled in red, were immediately observed in each tooth germ after ligation, and cervical loops at the sides of each split tooth germ gradually developed (Fig. 2a and Supplementary Movie 1). Secondary enamel knots, which are clusters of non-dividing cells (labelled in red), formed at a distance from the primary enamel knots after 3 days in organ culture (Fig. 2b and Supplementary Movie 2). We also investigated the expression patterns of *Shh* and *Fgf4*, which play important roles in the early development of tooth germs^1^,^2^, in ED14.5 natural and split tooth germs via *in situ* hybridisation (Fig. 2c). In natural tooth germs, *Shh* and *Fgf4* mRNA-positive cells were observed in the primary enamel knot after 1–2 days in organ culture (Fig. 3a). The *Shh* expression area stretched from the enamel knot to the crown area of the inner enamel epithelium after 3–4 days in organ culture (Fig. 2c). However, *Fgf4* mRNA-positive cells were strictly localised to the primary enamel knot after 1–2 days in organ culture and to the secondary enamel knot after 3–4 days in organ culture (Fig. 2c). The expression patterns of *Shh* and *Fgf4* mRNAs in the split tooth germ were identical to those in the natural tooth germ (Fig. 2c). Thus, the molecular mechanisms regulating early tooth development are remarkably similar in both split and natural tooth germs. For both ED14.5 natural and split tooth germs, we also investigated the expression pattern of *F-spondin* (*Spondin*), which is expressed in dental follicle cells, and of *Collagen, type I, alpha 1* (*Col1a1)*, which is expressed in dental mesenchyme and bone^31^ (Fig. 2d). After 6 days in organ culture, the expression of *Spondin* and *Col1a1* was detected in the dental follicles and in the dental mesenchyme and bone of the natural tooth germs and each split tooth germ, respectively. These results indicate that each split tooth germ developed into separated periodontal tissues after being subjected to mechanical ligation and showed the same gene expression patterns as a natural tooth.

### Re-regionalisation of tooth formation in split tooth germ development.

Previous studies have reported that the number of teeth is regulated by the reaction-diffusion waves of the expression of *Lef1*, an activator, and *Ectodin*, an inhibitor, in early tooth development^12^. To clarify the expression patterns in the tooth-forming field in split tooth germ development, we analysed the *Lef1* and *Ectodin* expression patterns in ED14.5 natural and split tooth germs by *in situ* hybridisation^22^,^23^. In the natural tooth germs, *Lef1* mRNA-positive cells were observed in the enamel knot and its neighbouring mesenchyme, whereas *Ectodin* mRNA-positive cells were observed primarily outside the *Lef1*-expression area^12^ (Fig. 2e). After 1 day in organ culture, each split tooth germ exhibited both a *Lef1*-expressing activation area and an *Ectodin*-expressing inhibition area surrounding the *Lef1*-expressing area (Fig. 2e). In addition, the gene expression intensities shown in Fig. 2f indicate that the period of the gene expression pattern of split tooth germs is half that of natural tooth germs. No significant differences in the intensity of mRNA expression of *Lef1* or *Ectodin* between split and natural tooth germs (Fig. 2g). These findings suggest that the tooth number and crown size are determined by the balance of expression of a specific activator and inhibitor and that they are moulded by the re-regionalisation of reaction-diffusion waves in the tooth-forming field.

### Transplantation of a split tooth germ into a tooth loss region.

Next, we performed micro-CT and tissue analyses to investigate whether a split tooth germ, ligated with an 8–0 nylon thread at ED14.5, erupted and became engrafted after transplantation into a tooth loss region (Fig. 3a). After 4–6 days in organ culture, a split tooth germ was transplanted in the correct orientation into a prepared socket of the upper first molar alveolar region in an adult murine tooth-loss transplantation model (Fig. 3a)^25^. During eruption, the alveolar bone at the socket gradually healed in the areas around the split teeth (Fig. 3b). Two months after transplantation, 71.9% of the split teeth (110/153) had formed the correct structures composed of enamel, ameloblasts, dentin, odontoblasts, dental pulp, alveolar bone, and periodontal tissues (Fig. 3c) and also had reached occlusal contact with the opposing mandibular first molar (Fig. 3d). These results indicate that transplanted split tooth germs can develop into two healthy teeth and can become engrafted in the alveolar bone with the appropriate formation of periodontal tissue.

### Functional regeneration by transplantation of a split tooth germ.

**Figure 4:**
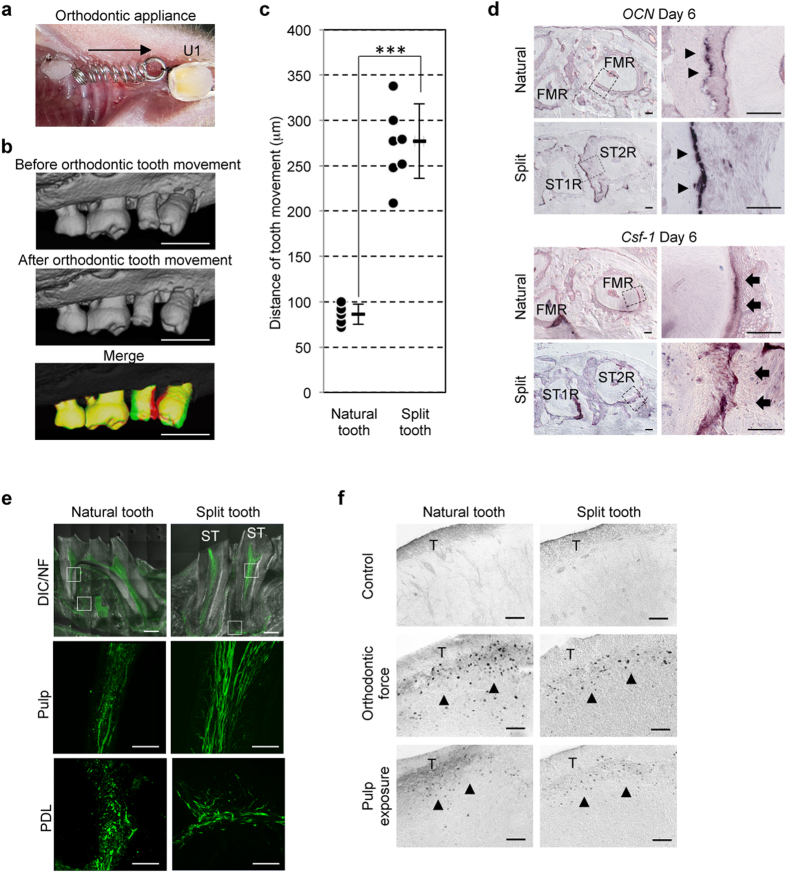
Functional regeneration via transplantation of a split tooth germ. (**a**) Intra-oral photograph after insertion of the appliance between the upper incisor (U1) and the anterior split tooth, or the upper second molar as a control. The anterior split tooth and the second molar moved in the anterior direction (arrow). (**b**) Micro-CT images before (upper) and after (lower) orthodontic movement, and superposition of micro-CT images at day 0 (red) and day 6 (green) of orthodontic movement. Scale bar, 500 μm. (**c**) The distances moved by a natural tooth and split teeth in response to orthodontic force were measured on day 0 and day 6 after orthodontic movement. The data are presented as the mean ± S.D.; n = 5 for natural teeth, n = 7 for split teeth. ^***^p < 0.001 (Student’s *t*-test). (**d**) Sections of natural teeth and split teeth were analysed via *in situ* hybridisation analysis for *Ocn* and *Csf-1* mRNA on day 6 of orthodontic movement. *Ocn* mRNA-positive cells (arrowhead) and Csf-1 mRNA-positive cells (arrow) are indicated. FMR, first molar root; STR, split tooth root. Scale bar, 100 μm. (**e**) Nerve fibres in the pulp and the PDL in the natural tooth (upper) and split teeth (lower) were analysed via immunohistochemistry using antibodies specific for NF (green). ST, split tooth. Scale bar, 50 μm. (**f**) Analysis of c-Fos-immunoreactivity in the medullary dorsal horns of mice with a natural tooth (upper) or a split tooth (lower) after 0 hours (no force, control; left) or 2 hours of application of orthodontic force (middle) or pulp exposure (right). c-Fos protein (arrowhead) was detectable after application of either stimulus to both natural (left) and split (right) teeth at 60 days post-transplantation (lower). T, spinal trigeminal tract. Scale bar, 100 μm.

For tooth regenerative therapy, the split tooth must be able to restore functions such as mastication^32^ and normal responses to mechanical stress^33^ and noxious stimuli^34^, including communication with both the oral and central nervous system. To analyse the function of the PDL of the engrafted split teeth, we applied orthodontic force using a 10 gf Ni-Ti closed-coil 50 days after transplantation (Fig. 4a) and observed the movement of the natural and split teeth (Fig. 4b). The natural first molars moved 86.0 ± 11.0 μm, and the split teeth moved 277.0 ± 41.1 μm in response to the application of orthodontic force on day 6 (Fig. 4b,c). The split teeth moved significantly farther in response to orthodontic force because the split teeth have a single-root shape, in contrast to the natural tooth, which have a multiple-root shape (Fig. 4b,c). These results suggested that split teeth have strong potential for orthodontic movement in response to mechanical force. During tooth movement, colony-stimulating factor-1 (*Csf-1*) mRNA-positive cells, which were used as a marker of osteoclastogenesis, and osteocalcin (*Ocn*) mRNA-positive osteoblasts were observed on the compression and tension sides, respectively^25^,^33^,^35^ (Fig. 4d). This finding demonstrated that the PDL of the split teeth successfully mediates bone remodelling through the proper localisation of osteoclasts and osteoblasts in response to orthodontic force. We evaluated the innervation of the pulp and the PDL, which plays essential roles in maintaining the homeostasis and function^34^ of natural and split teeth, at 50 days post-transplantation, by immunohistochemistry using antibodies specific for neurofilament (NF) (Fig. 4e). Neurofilament (NF)-immunoreactive nerve fibres were detected in the pulp and the PDL of the natural and engrafted split teeth. Finally, after exposure to noxious stimuli such as orthodontic force and pulp exposure, we evaluated the expression of c-Fos immunoreactive neurons in the superficial layers of the medullary dorsal horn^34^. c-Fos was observed in both the natural and split teeth 2 hours after stimulation (Fig. 4f). These results indicate that the engrafted split teeth responded normally to noxious stimuli via their nerves *in vivo* and maintained the same communication with the central nervous system as a natural tooth.

## Discussion

Here, we demonstrated the generation of multiple teeth by splitting tooth germs based on a re-regionalisation of the tooth-forming field, which is controlled by a reaction-diffusion model under artificial manipulation. The split teeth erupted into the oral cavity and restored physiological tooth functions, including mastication, PDL function and responsiveness to noxious stimuli. This study presents a novel tooth regenerative technology using split tooth germs and re-regionalisation of the tooth-forming field in response to artificial mechanical force.

The tooth-forming field and the basic pattern of dentition, including the incisors and molars in mice, are regulated by the distribution and gradient of specific morphogen during the initial phase of tooth germ development^3^,^36^. This molecule-based patterning, including rostral-caudal patterning through the expression of *Lhx6/7* and *Gsc* and proximal-distal patterning through the expression of *Bmp4* and *Fgf8*, contributes to the regionalisation of tooth germ formation and the determination of tooth types^3^. It is also thought that the number of teeth is regulated by these reaction-diffusion waves of gene expression, including *Lef1* or *Ectodysplasin* (activators) and *Ectodin* (an inhibitor)^12^,^37^. Tooth morphology, in which macro-patterning determines crown size and tooth length, is also regulated by this reaction-diffusion model, based on a developmental program that is programmed in the dental mesenchyme^13^. Using our *in vitro* experimental model of the tooth-forming field, we previously showed that the spatiotemporal regulation of epithelial cell proliferation and *Shh* expression are closely involved in the determination of tooth macro-morphology and constitute the molecular basis of tooth size determination^38^. In this study, we demonstrated the successful generation of split tooth germs, which leads to the creation of two signalling centres that are both designated as primary enamel knots, in response to mechanical force. The tooth-forming field is defined by *Lef1* and *Ectodin* expression, and the regulation of crown width is defined by *Shh* and *Fgf4* expression, which facilitate the proper development of the split tooth germs. These findings indicate that tooth macro-morphology, including the number and size of teeth, can be determined by re-regionalisation of the tooth-forming field, which is regulated by reaction-diffusion waves of activators and inhibitors.

Regulation of the number and position of cusps, which underlies the micro-patterning of teeth, is thought to be closely involved in the formation of the secondary enamel knot^39^. A previous study reported that a reaction-diffusion model for spatiotemporal cusp patterning in the tooth developmental process was regulated by interactions among the cusp-formation activator *Fgf4* and the inhibitors *BMPs* and *Shh*^7^,^14^,^39^. It was also demonstrated that the cusp numbers in bioengineered teeth were correlated with the crown width, which was regulated by the contact area between the epithelial and mesenchymal cell layers^38^. These findings suggest that the micro-patterning of cusp formation is regulated after tooth size is determined (*i.e*., after macro-patterning) by the frequency and wave length in the reaction-diffusion model^38^. In the current study, the cusp number of the split tooth germ was regulated by *Fgf4* expression, which designated it as a secondary enamel knot, and was correlated with the crown width of the split tooth. The crown width of the split tooth was artificially defined by the re-regionalisation of the tooth-forming field through reaction-diffusion waves of *Lef1* and *Ectodin* expression. These findings suggest that the micro-patterning of cusp morphology can be redefined in a restricted area by reaction-diffusion after re-regionalisation of the tooth-forming field.

Conventionally, dental treatments with artificial materials such as dental bridges, dentures and dental implants have been widely used after tooth loss^19^,^20^. Although these artificial therapies are effective, the development of a novel biologically based dental therapy is sought to restore the physiological functions of teeth, including sufficient masticatory performance, biological cooperation with the periodontal tissues and afferent responsiveness to noxious stimuli in the maxillofacial region^21^. Recently, the first reports of fully functional bioengineered tooth replacements with the correct tissue orientation, masticatory potential, responsiveness to mechanical stress and perceptive potential following the transplantation of a bioengineered tooth germ into a tooth-loss region were published^25^,^40^. The progress that has been made in tooth regenerative technology is remarkable; however, suitable cell sources that prevent immunological rejection must be identified for future clinical applications of tooth-replacement therapy^4^. In the present study, we developed a novel mechanical ligation method to increase the number of transplantable tooth germs. Following transplantation, the split tooth germs erupted into tooth-loss regions and restored physiological tooth functions, including mastication, periodontal ligament function through bone remodelling, and proper responsiveness to noxious stimuli. These findings indicate that the transplantation of split tooth germs has strong potential as a practical tooth regenerative therapy that can be used to treat tooth loss caused by both congenital syndromes and non-syndromic diseases.

In conclusion, our study demonstrates a new functional tooth regeneration technology utilising split tooth germs through re-regionalisation of the tooth-forming field by applying mechanical force. Further studies to identify tooth germs at the appropriate developmental stage for use and to optimise the splitting procedure in humans are necessary for the future clinical application of split tooth germ transplantation.

## Methods

### Animals

C57BL/6 mice were purchased from SLC Inc. (Shizuoka, Japan). B6.B6D2-Tg(Fucci)596Bsi mice were obtained from the RIKEN Bioresource Center (Tsukuba, Japan). R26-H2B-EGFP mice were generously provided by Dr. Toshihiko Fujimori (National Institute for Basic Biology, Aichi, Japan). All mouse care and handling protocols complied with the NIH guidelines for animal research, and all experimental procedures were performed in accordance with the protocols approved by the Tokyo University of Science Animal Care and Use Committee (Permit Number: N13009).

### Splitting of a tooth germ by ligation

First molar tooth germs were exenterated from the mandibles of ED14.5 mice. To determine the crown-root direction, the dental lamina, which is a transparent band and gives rise to the primordia of the enamel organs of the teeth, is used as a reference point. Each isolated tooth germ was ligated using a fine thread (8–0 nylon surgical suture; Natsume, Tokyo, Japan) by manipulating the thread around the mesiodistal centre of the tooth germ and slowly tightening the thread until the two regions were constricted into a bottleneck shape. To determine the ligation force value, we strictly defined an inner diameter of 50 μm with an 8–0 nylon thread. The ligated tooth germs were incubated for 5 min at 37 °C, placed on a cell culture insert (0.4 μm pore diameter; Corning, NY, USA), and then further incubated at 37 °C for 6 days in *in vitro* organ culture, as described previously^26^,^41^.

### Subrenal Capsule Transplantation

After 6 days of cultivation, the split tooth germs were transplanted into a subrenal capsule for 30 days using 7–8 week-old mice as the host, as described previously^26^,^40^.

### Histochemical Analysis and Immunohistochemistry

Histochemical tissue analyses were performed as described previously^26^. Tissue sections (5–10 μm) were stained with haematoxylin-eosin and observed using an Axio Imager A1 microscope (Carl Zeiss, Oberkochen, Germany) fitted with an AxioCAM MRc5 camera (Carl Zeiss). To prepare the tissues for immunohistochemistry, mice under deep anaesthesia were perfused transcardially with 4% paraformaldehyde in PBS (–). Tissues were then removed and further post-fixed for 2–16 h at 4 °C. After fixation, the tissues were prepared as described previously^26^. For fluorescence immunohistochemistry, the sections (35 μm) were incubated with the primary antibody against the neurofilament SMI312 (1:1,000, mouse, Abcam, Cambridge, MA, USA). The primary antibody was detected using a highly cross-adsorbed Alexa Fluor®594 goat anti-rabbit IgG (H+L) (1:200, Life Technologies, Carlsbad, CA, USA). Fluorescence microscopy images were captured using a laser confocal microscope (LSM 780, Carl Zeiss). Enzyme immunohistochemistry was performed as described previously^42^. Sections (50 μm) of the medullary dorsal horn in the brainstem region were incubated with anti-c-Fos Ab (1:10,000, Santa Cruz Biotechnology, Dallas, TX, USA). The sections were then immunostained with peroxidase-labelled goat anti-rabbit IgG (1:300, Cappel Laboratories, Cochranville, PA, USA) and PAP immune complex (1:3,000, Cappel). The stained sections were observed on an Axiovert microscope (Carl Zeiss) equipped with an AxioCAM MRc5 camera (Carl Zeiss).

### *In situ* hybridisation

*In situ* hybridisation was performed using 10-μm frozen sections, as described previously^26^. Digoxigenin-labelled probes for specific transcripts were prepared via PCR, and the primers were designed using published sequences (GenBank ID; *Shh*: NM_009170, *Fgf4*: NM_010202, *F-spondin* (*Spondin*): NM_145584, *Collagen, type I, alpha 1* (*Col1a1*): NM_007742, *Ectodin*: NM_025312, *Lef1*: NM_010703, *Colony-stimulating factor 1* (*Csf-1*): NM_007778, *Osteocalcin* (*Ocn*): NM_007541).

### RNA preparation and real-time PCR

Total RNA was isolated from natural and split tooth germs from ED14.5 mice mandibular at one day after incubation using RNAeasy Micro Kit (QIAGEN) according to the manufacturer’s protocol. cDNAs were synthesised from aliquots of total RNA using the PrimeScript II 1st strand cDNA Synthesis kit (Takara Bio). mRNA expression levels were determined using SYBR Premix Ex Taq II (Takara Bio), and the products were analysed with a QuantStudio 12K Flex (Applied Biosystems, CA, USA). Specific primers for *Lef1* (forward, 5-cgatccccagaaggagaagat-3; reverse, 5-gggatgatttcggactcgtta-3) and *Ectodin* (forward, 5-GAATCAAGCCAGGAATGGAG-3; reverse, 5-GTATTTGGTGGACCGCAGTT-3) were used for real-time PCR.

### Transplantation of a split tooth germ

The upper first molars of 4-week-old C57BL/6 mice were extracted under deep anaesthesia. Transplantation was carried out 3 weeks later, to allow natural healing of the sockets. Before transplantation, we confirmed that there were no remaining tooth root components and/or teeth developing from the root components, by using micro-CT. The transplantation of split tooth germs followed the procedure in our previous reports^25^,^41^. Following repair, an incision of approximately 1.5 mm in length was made through the oral mucosa at the extraction site using surgical knives to access the alveolar bone. A fine pin vice (Tamiya, Shizuoka, Japan) was used to create a socket of approximately 1.0–1.5 mm in diameter in the exposed alveolar bone surface. Just before transplantation, we removed the collagen gel from the split tooth germ in the *in vitro* organ culture and marked the top of the dental epithelium with a vital staining dye, such as methylene blue, to ensure the correct crown-root direction of the explants. The explants were then transplanted into the prepared socket. The incised oral mucosa was sutured with 8–0 nylon (8–0 black nylon 4 mm 1/2R, Bear Medic Corp., Chiba, Japan), and the surgical site was cleaned.

### Microcomputed tomography (Micro-CT) measurements

Radiographic imaging was performed using X-rays and a micro-CT device (R_mCT, Rigaku, Tokyo, Japan) with exposures set at 90 kV and 150 mA. Micro-CT images were captured using i-view R software (Morita, Kyoto, Japan).

### Live imaging of a split tooth germ from a transgenic mouse embryo

A time-lapse movie of an ED14.5 embryo obtained by crossing B6.B6D2-Tg(Fucci)596Bsi and Rosa26H2B-EGFP mice was acquired. Molar tooth germs were exenterated from the mandibles of an ED14.5 embryo and ligated using an 8–0 nylon thread, as described above. To hold the embryo in position during time-lapse movie acquisition, the tooth germs were placed in a collagen type I-A (Nitta gelatin, Osaka, Japan) gel drop on glass bottom dishes (IWAKI, Tokyo, Japan) coated with collagen type I-P (Nitta gelatin), and the dishes were then filled with culture medium. The time-lapse imaging of the split tooth germs was captured using a confocal laser microscope (LSM 780; Carl Zeiss) in a CO_2_ incubator while the split tooth germs were cultured in an atmosphere of 5% CO_2_ at 37 °C. Images taken at 35–40 min intervals were analysed using Imaris software (Bitplane, Zurich, Switzerland).

### Orthodontic tooth movement and biomechanics

The orthodontic appliance was composed of a 10 gf Ni-Ti closed-coil spring that continuously applied a force of 10 gf between approximately 0.1 and 5.0 mm. This appliance was inserted between the upper incisors and the anterior split teeth of the mice. The appliance was tied with a piece of 8–0 nylon (Natsume) to the lateral incisors and fixed with resin at the alveolar bone level. The posterior end of the appliance was tied to the anterior split tooth with a piece of 8–0 nylon. After 4 days, the appliance was removed, and the distance between the two split teeth was maintained by fixing resin between both crowns. We repeated these experiments 2–3 times. In the control group, orthodontic force was applied in the mesial direction to the upper first molar of 7-week-old normal C57BL/6 mice in the same manner as the experimental group. Serial sections were analysed by *in situ* hybridisation for macrophage colony-stimulating factor (*Csf-1*) and osteocalcin (*Ocn*) mRNAs as previously described^25^,^40^. We measured the orthodontic movement distance between the anterior and posterior split tooth crowns using i-view R (Morita, Kyoto, Japan).

### Statistical analysis

The data are presented as the mean ± standard deviation (S.D.). We used Dunnett’s test, Steel’s test a two-tailed Student’s *t*-test and Tukey test to determine the *p*-values for statistical significance. *p*-values less than 0.05 was considered to be statistically significant. The analyses were performed using JMP (version 10.0; SAS Institute Inc., NC, USA).

## Additional Information

**How to cite this article**: Yamamoto, N. *et al*. Functional tooth restoration utilising split germs through re-regionalisation of the tooth-forming field. *Sci. Rep*. **5**, 18393; doi: 10.1038/srep18393 (2015).

## Supplementary Material

Supplementary Information

Supplementary Movie S1

Supplementary Movie S2
