# Supplementary material for: Functional tooth restoration utilising split germs through re-regionalisation of the tooth-forming field
Source: Sci Rep. 2015 Dec 17;5:18393. doi: 10.1038/srep18393 (PMC4682098; doi:10.1038/srep18393)
Supplement: Supplementary Information [file srep18393-s1.pdf]

SUPPLEMENTARY INFORMATION

Functional tooth restoration utilising split germs through re-regionalisation of the tooth-forming field

Naomi Yamamoto[1, 2], Masamitsu Oshima[3, 4], Chie Tanaka[2], Miho Ogawa[4, 5, 6], Kei Nakajima[2, 7], Kentaro Ishida[4, 8], Keiji Moriyama[1] & Takashi Tsuji[2, 4, 5, 6*]

[1]*Department of Maxillofacial Orthognathics, Tokyo Medical and Dental University, Bunkyo-ku, Tokyo, 113-8510, JAPAN*

[2] *Department of Biological Science and Technology, Graduate School of Industrial Science and Technology, Tokyo University of Science, Noda, Chiba, 278-8510, JAPAN*

[3] *Department of Oral Rehabilitation and Regenerative Medicine, Graduate School of Medicine, Dentistry and Pharmaceutical Sciences, Okayama University, Okayama, 700-8525, JAPAN*

[4] *Research Institute for Science and Technology, Tokyo University of Science, Noda, Chiba, 278-8510, JAPAN*

[5] *Organ Technologies Inc., Tokyo, 108-0074, JAPAN*

[6] *RIKEN Center for Developmental Biology, Kobe, Hyogo, 650-0047, JAPAN*

[7]*Department of Clinical Pathophysiology, Tokyo Dental College, Chiyoda-ku, Tokyo, 101-0061, JAPAN*

[8]*Department of Physics and Mathematics, College of Science and Engineering, Aoyama Gakuin University, Sagamihara, Kanagawa, 252-5258, JAPAN*

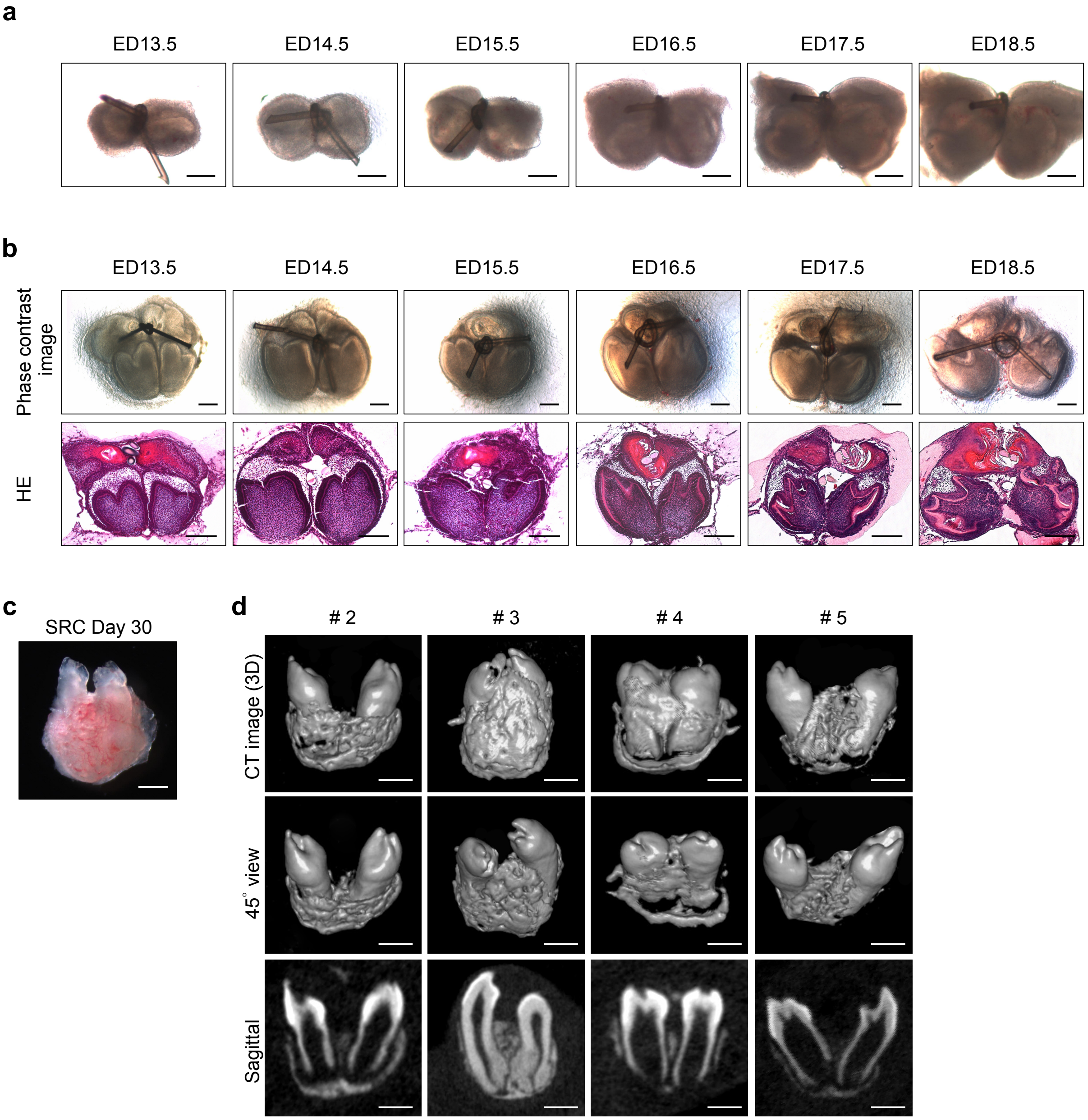
a
ED13.5
ED14.5
ED15.5
ED16.5
ED17.5
ED18.5
b
ED13.5
ED14.5
ED15.5
ED16.5
ED17.5
ED18.5
Phase contrast image
HE
c
SRC Day 30
d
2
3
4
5
CT image (3D)
45° view
Sagittal

Supplementary Figure 1: Assessment of a split tooth germ

(a) Phase-contrast images of ED13.5, ED14.5, ED15.5, ED16.5, ED17.5, and ED18.5 split tooth germs immediately after ligation. Scale bar, 100 μm.

(b) Phase-contrast images of ED13.5, ED14.5, ED15.5, ED16.5, ED17.5, and ED18.5 split tooth germs (upper) and HE section images of each split tooth germ after 6 days of organ culture. Scale bar, 100 μm.

(c) Photograph of split teeth on day 30 after subrenal capsule transplantation (SRC). Scale bar, 500 μm.

(d) Micro-CT images of the external surface area (upper), 45º view (centre) and cross section (lower) of natural teeth 30 days after SRC transplantation. Scale bar, 500 μm.

Supplementary Movie 1: Live imaging of a split tooth germ during early development (I).

A time-lapse movie of a split tooth germ using transgenic mouse embryos was recorded. The recording was made immediately after ligation, during which the primary enamel knot (labelled in red) develops. Red fluorescence indicates the G0/G1 phase area. Scale bar, 100 μm.

Supplementary Movie 2: Live imaging of a split tooth germ during early development (II).

A time-lapse movie of a split tooth germ using transgenic mouse embryos was recorded. The recording was made after 2.5 days in organ culture. The secondary enamel knot (labelled in red) appears during the movie. Red fluorescence indicates the G0/G1 phase area. Scale bar, 100 μm.
